# Site-directed mutagenesis of cysteine residues alters oxidative stability of fetal hemoglobin^☆^

**DOI:** 10.1016/j.redox.2018.08.010

**Published:** 2018-08-22

**Authors:** Karin Kettisen, Michael Brad Strader, Francine Wood, Abdu I. Alayash, Leif Bülow

**Affiliations:** a Pure and Applied Biochemistry, Department of Chemistry, Lund University, Lund 22362, Sweden; b Laboratory of Biochemistry and Vascular Biology, Center for Biologics Evaluation and Research, Food and Drug Administration, Silver Spring, MD 20993, USA

**Keywords:** CO, carbon monoxide, DTT, 1,4-dithiothreitol, Hb, hemoglobin, HbA, adult hemoglobin, HbF, fetal hemoglobin, OBC, oxygen binding curve, SOD, superoxide dismutase, XIC, extracted ion chromatogram, Fetal hemoglobin, Site-directed mutagenesis, Cysteine, Oxidation, Hydrogen peroxide, Protein electron transfer

## Abstract

Redox active cysteine residues including βCys93 are part of hemoglobin's “oxidation hotspot”. Irreversible oxidation of βCys93 ultimately leads to the collapse of the hemoglobin structure and release of heme. Human fetal hemoglobin (HbF), similarly to the adult hemoglobin (HbA), carries redox active γCys93 in the vicinity of the heme pocket. Site-directed mutagenesis has been used in this study to examine the impact of removal and/or addition of cysteine residues in HbF. The redox activities of the recombinant mutants were examined by determining the spontaneous autoxidation rate, the hydrogen peroxide induced ferric to ferryl oxidation rate, and irreversible oxidation of cysteine by quantitative mass spectrometry. We found that substitution of γCys93Ala resulted in oxidative instability characterized by increased oxidation rates. Moreover, the addition of a cysteine residue at α19 on the exposed surface of the α-chain altered the regular electron transfer pathway within the protein by forming an alternative oxidative site. This may also create an accessible site for di-sulfide bonding between Hb subunits. Engineering of cysteine residues at suitable locations may be useful as a tool for managing oxidation in a protein, and for Hb, a way to stave off oxidation reactions resulting in a protein structural collapse.

## Graphical abstract

**Figure:**
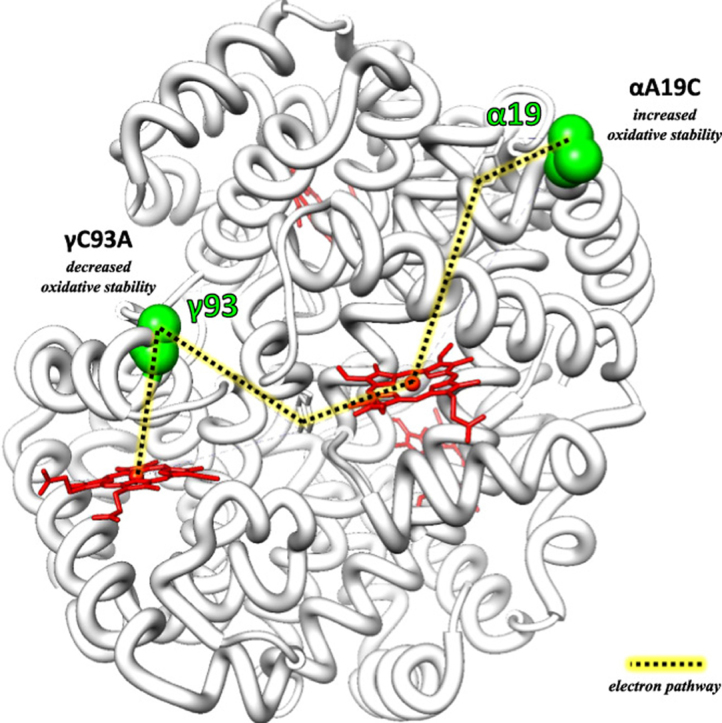

## 1 Introduction

Human fetal hemoglobin (HbF) is the main oxygen carrying protein present during the fetal development stages and up until six months after birth [1]. Similar to human adult hemoglobin (HbA), it is a tetrameric protein that harbors two α-chains, but these are paired with two γ-chains instead of the β-chains of HbA. The gene encoding the γ-chain is duplicated and gives rise to two variants: Aγ (HBG1) and Gγ (HBG2). The only difference between the two variants is located at position 136 which is either an alanine or a glycine. The γ-chain and the β-chain both consist of 146 amino acid residues but differ in their sequences at 39 or 40 positions, for Gγ and Aγ respectively, resulting in differences in some properties between HbA and HbF [2]. The oxygen affinity is higher for HbF than HbA. This has been reported to be due to residues at positions γ1, γ5, γ43 and γ143 [3], [4], [5], resulting in lower binding of 2,3-diphosphoglycerate, which acts as an allosteric regulator. Residues in the N-terminal region (Helix A) of the γ-chain have also been shown to contribute to HbF tetramer stabilization compared to HbA, which is reflected by a 70-fold lower tetramer-dimer dissociation constant [6]. The solubility of HbF is higher than HbA, possibly due to position γ22 which is an aspartate instead of glutamate as in β-chain, and this property may contribute to the reported antisickling ability of HbF [7]. Furthermore, HbF has a higher stability in alkaline solution. The mechanism is not fully known, but it was thought to be related to internal amino acids. Positions βCys112 and βTyr130, which can be ionized, are replaced in the γ-chain to γThr112 and γTrp130; thereby reducing destabilization of the αγ contacts and attraction of water into the non-polar interior at high pH [8]. It was also suggested that the source of this property appeared to be located in the C-terminal of the γ-chain [9]. However, mutants of the proposed residues could not directly verify this hypothesis [10].

It is clear that amino acid substitutions can have biological effects on the overall functions of the Hb molecule. Thus, protein engineering strategies such as site-directed mutagenesis can be utilized to develop a better understanding and control oxidative reactions within the protein. The most significant oxidation-prone sites in HbA when reacting with H_2_O_2_ are the cysteine residues at β93 and β112 [11]. These reactions ultimately lead to structural collapse of the heme pocket, heme release, and eventually breakdown of the protein [11]. Cys93 is present in both the β- and γ-chain, and this site has been confirmed to be part of the oxidative hotspot in HbF through quantitative mass spectrometry [12]. Understanding the oxidation patterns within the protein may enable rational engineering to regulate this destructive pathway. In a study comparing mouse Hb with either two cysteine residues in the β-chain, βCys13 and βCys93, or only a single cysteine at βCys93, it was found that the presence of βCys13 lowered metHb formation during H_2_O_2_ exposure [13]. Modified cysteine content within a subunit appears thus to enable alternative electron transfer pathways in the Hb molecule.

In HbF there is one cysteine residue per subunit, at positions αCys104 and γCys93. Three mutants of HbF were created in this study to explore the oxidative influence of cysteine residues in HbF. α19 Ala→Cys (αA19C), γ93 Cys→Ala (γC93A) and double mutant αA19C/γC93A. None of these mutations have been found as natural mutations according to the database of Hb variants [14]. However, substitutions at these sites to other residues are represented. Hb J-Tashikuergan α19 Ala→Glu, was found in healthy males with no hematological effect reported [15]. Hb F-Monserrato-Sassari γ93 Cys→Arg appeared in a healthy baby and was found to have increased oxygen affinity and a slight decrease of both Bohr effect and cooperativity [16]. More examples of natural mutations at position Cys93 are represented in β-chain of HbA, such as: Hb Okazaki β93 Cys→Arg, which was found in a healthy male and showed increased oxygen affinity and molecular instability [17]; Hb Fort Dodge β93 Cys→Tyr was reported in a healthy female with no clinical or hematological effects [18]; Hb Riesa β93 Cys→Ser was found in a diabetic male but hematologically and clinically silent [19]; Hb Santa Giusta Sardegna β93 Cys→Trp was found in healthy males with increased Hb content, and the Hb was determined to exhibit increased oxygen affinity, decreased cooperativity and reduced stability [20]. In the two cases of decreased stability the cysteine was replaced by more bulky amino acids. It appears therefore that some mutations at α19 and γ93 have the potential to produce stable Hb proteins, but the effect in regard to oxidative stability is left to be explored. In this study, we have investigated oxidative influence of modified cysteine content in recombinant human HbF.

## 2 Materials and methods

### 2.1 Recombinant HbF production

**Fig. 1:**
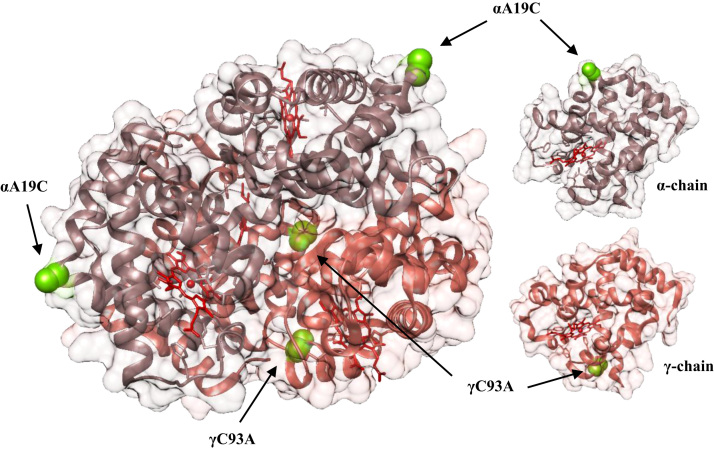
Structure of HbF (PDB code: 4MQJ) with α-chains in purple and γ-chains in pink. Residues targeted by site-directed mutagenesis are shown in green, and heme groups are colored red.

The gene expressing wildtype recombinant HbF (rHbF) was previously inserted into the pETDuet-1 plasmid from Novagen [21]. Site-directed mutagenesis was used to mutate the rHbF gene at specific sites of interest, and the method has been described previously [22]. Three mutants were formed by; 1) substitution of an alanine residue for cysteine at position 19 in the α-chain (αA19C); 2) substitution of a cysteine residue for alanine at position 93 in the γ-chain (γC93A); and 3) a mutant with both previous substitutions (αA19C/γC93A). The locations of the mutation sites are displayed in Fig. 1. Site-directed mutagenesis primers were acquired from Integrated DNA Technologies (IDT, Germany). Mutated plasmids were sequenced at GATC Biotech (Germany) by Sanger sequencing, and transformed into *Escherichia coli* BL21 (DE3) for expression.

### 2.2 Production and purification of recombinant HbF

The *E. coli* containing rHbF, αA19C, γC93A or αA19C/γC93A were all cultivated in the same manner and the expression and purification procedures were based on previous protocols [21], [22], [23], with some modifications. 500 ml Terrific Broth media in 2 l Erlenmeyer flasks were inoculated with overnight starter culture and immediately induced with 0.1 mM isopropyl β-_D_-1-thiogalactopyranoside (IPTG, Saveen & Werner) and supplemented with 0.24 mM δ-aminolevulinic acid (Sigma-Aldrich). The cultures were briefly bubbled with carbon monoxide gas (CO) and then cultivated overnight in 30 °C at 150 rpm. CO gas was bubbled into the cultures directly after terminating the cultivation to stabilize the Hb in CO-liganded form. The cells were processed according to aforementioned protocols up until removal of host contaminating proteins, which was performed by liquid chromatography on an ÄKTA Avant 25 system (GE Healthcare) involving two ion exchange purification steps. All buffers were prepared with ultrapure water (Purelab ultra, ELGA Veolia Water solutions and Technologies, Väsby, Sweden), filtered and degassed before use. The first step involved a cation exchange column, packed with 65 ml Capto S media (GE Healthcare) in a HiScale 26/20 column (GE Healthcare). The flow velocity was 153 cm/h. The Hb bound to the column pre-equilibrated with 10 mM sodium phosphate (NaP) buffer pH 6.0 and was released by isocratic elution with 70 mM NaP buffer pH 7.2. After exchanging the sample buffer to 20 mM Tris-HCl pH 8.3 using a Sephadex G-25 desalting column (GE Healthcare), the second chromatography step was anion exchange with a HiTrap Q HP 5 ml column (GE Healthcare). A linear gradient elution to 50 mM sodium phosphate buffer pH 7.2 supplemented with 100 mM NaCl resulted in pure fractions of Hb. Additionally, the αA19C and αA19C/γC93A samples were supplemented with 10 mM DTT (1,4-dithiothreitol, Sigma-Aldrich) and incubated 20 min on ice before being applied onto the Q HP column in order to reduce possible disulfide bond formation between the polypeptide chains. The Hb solutions were finally concentrated by centrifugation with Vivaspin columns (30,000 MWCO, Sartorius) and snap-frozen in liquid nitrogen in the CO-liganded state and stored at − 80 °C. The purity was confirmed by sodium dodecyl sulfate polyacrylamide gel electrophoresis (SDS-PAGE) and the gel was analyzed densitometrically using a Molecular Imager Gel Doc XR System, with Quantity One software (Bio-Rad Laboratories). The amino-terminal Met residues were removed (> 95%) as determined by mass spectroscopic measurements.

### 2.3 Biophysical characterization: cysteine quantification, light scattering and oxygen equilibrium curve analysis

Ellman's reagent (5,5′-dithio-bis-(2-nitrobenzoic acid), Thermo Scientific) was used to quantify the cysteine content in the Hb samples. The reaction buffer (100 mM NaP buffer pH 8.0 + 1 mM EDTA), Ellman's reagent (71.4 µg/ml) and Hb samples were mixed and incubated at room temperature for 15 min before measurement of absorbance at 412 nm, according the manufacturer's protocol. The sample absorbance was compared to a standard curve prepared with L-cysteine (Sigma-Aldrich). The purified proteins were also analyzed by dynamic light scattering (DLS) in a Zetasizer Nano S instrument (Malvern). The Hb samples were diluted to 0.5, 1.5 and 5.0 mg/ml in 50 mM NaP buffer pH 7.2, and measured at 20 °C. First without DTT, and then incubated with addition of 10 mM DTT for 10 min and measured again. Oxygen equilibrium curves of all recombinant proteins were recorded on a Hemox Analyzer (TCS Scientific, New Hope, PA) in 10 mM potassium phosphate buffer pH 7.4 supplemented with 100 mM NaCl at 37 °C, as described previously [24].

### 2.4 Autoxidation kinetics

Hb samples were thawed, kept on ice, and fully converted into the ferrous O_2_-liganded form by exposure to a continuous stream of oxygen gas under bright light. The conversion from CO-liganded state to the oxy-form was confirmed by spectral shift resulting in peak maxima at 415, 540 and 576 nm, followed by buffer exchange to 100 mM NaP buffer pH 7.4 using a Sephadex G-25 desalting column. The concentration of Hb was confirmed by addition of sodium dithionite (Sigma-Aldrich) to a small portion of the sample, converting it to the deoxy-form, and using the millimolar extinction coefficient 133 mM^−1^ cm^−1^ at absorbance peak 430 nm. The autoxidation of 20 μM Hb was monitored with Cary60 UV–vis spectrophotometer (Agilent Technologies) at 25 °C during 24 h, with or without addition of superoxide dismutase (SOD) and catalase at concentrations of 4.6 U/ml and 414 U/ml, respectively. The rate of autoxidation was determined by following the decrease in absorbance at 576 nm over time. The data were fitted to a single exponential equation.

### 2.5 Stopped flow kinetics

The Hb samples were thawed, mixed with 1:1.5 excess of potassium ferricyanide (K_3_[Fe(CN)_6_] (Sigma-Aldrich), incubated on ice, and exposed to visible light for complete conversion to the ferric form (Fe^3+^). The samples were then loaded on Sephadex G-25 desalting columns equilibrated with 40 mM NaP buffer pH 7.2 to remove the surplus of potassium ferricyanide. The concentration of Hb was determined as described before and sample solutions of 20 μM Hb were prepared. H_2_O_2_ solutions at concentrations of 200, 400, 400, 600, 800 and 1000 µM were prepared freshly the same day with ultrapure water, kept on ice and away from light until the start of the experiment. The oxidation reaction of ferric Hb and H_2_O_2_ were performed in a stopped-flow experimental setup, previously described by Ratanasopa et al. [12]. Briefly, the Hb samples and H_2_O_2_ were mixed rapidly with the RX-2000 Rapid Mixing Stopped-Flow Accessory (Applied Photophysics, United Kingdom) and the reaction was followed at 405 nm in a Cary 60 UV–vis spectrophotometer (Agilent Technologies). The data were fitted to a double exponential equation. The obtained rate constants were plotted against H_2_O_2_ concentration and fitted to a linear equation.

### 2.6 Quantitative mass spectrometry

178 µM of each recombinant protein recombinant proteins (rHbF, αA19C, γC93A, and αA19C/γC93A) was converted to the oxy-form treated with incremental doses (0, 5×, 10×) of H_2_O_2_ and then incubated overnight in 20 mM NaP pH 7.4 at 25 °C to replicate oxidative conditions. All samples were then processed and treated with trypsin as previously described [25]. LC/MS/MS analysis and targeted quantitative proteomics were utilized to identify and quantify oxidative modifications in the Hb samples. All data were performed in triplicate on a Q-Exactive mass spectrometer coupled to a Proxeon HPLC system. MS/MS spectra were initially searched against the human database supplemented with the above recombinant sequences using both Protalizer software and the Mascot database search algorithm to confirm sequence identity and identify unmodified and oxidized version of hotspot peptides. Search parameters specified for detecting variable modifications including oxidation of methionine (+ 16 Da), cysteine (+ 48 Da) and tyrosine oxidation (+ 16 Da). Because all experimental samples were denatured and treated with iodoacetamide prior to trypsinization, an additional static search involving carbamidomethylation of cysteine was included to identify all unoxidized cysteine (not oxidized in presence of H_2_O_2_). The precursor ion mass tolerance was ± 10 ppm and the fragment ion mass tolerance was ± 0.025 Da. Extracted ion chromatograms of modified (and unmodified) tryptic peptides were used to quantify oxidative differences. For relative quantification, the ratio of each oxidized hotspot peptide was calculated based on the sum of the extracted ion chromatogram (XIC) peak area of all forms (oxidized and unmodified) to be 100%.

### 2.7 Heme loss kinetics

Heme loss from the different recombinant Hbs was examined by utilizing heme scavenger apomyoglobin H64Y/V67F (apoMb) according to the method developed by Hargrove et al. [26]. The experimental procedure was adopted from Silkstone et al. [27]. Hb proteins were completely converted into the ferric form as previously described and incubated at 37 °C together with excess of apoMb in 100 mM NaP buffer pH 7.2, supplemented with 150 mM sucrose. The final concentrations of Hb and apoMb were 2.5 µM and 30 µM, respectively. The reaction was monitored by scanning the spectra of the Hbs in the range 675–375 nm in a Cary60 UV–vis spectrophotometer (Agilent Technologies). The rate of heme release from the α-chain was determined over the course of 12 h. The data were fitted to a double exponential equation where the slower rate described the rate of heme loss from the α-chain. The rate of release from the γ-chain was faster, and observed by recording the spectra every 30 s for 20 min. The data were fitted to a single exponential equation and the rate was determined for the γ-chain.

### 2.8 Data and statistical analysis

All experimental reactions were replicated a minimum of three times (n = 3), and mean values and standard deviations were calculated. The Microsoft Excel Solver program was used to fit the time courses obtained to exponential functions. Statistical significance (P < 0.05) was determined by unpaired *t*-tests.

## 3 Results

The correctness of the DNA sequences of the wildtype and the three mutants were confirmed by Sanger sequencing. All recombinant proteins were subsequently expressed in *E. coli* and purified successfully. The obtained yields 0.7–1.1 mg Hb/g cells and 6.0–9.3 mg Hb/l culture were well within previously reported range of wildtype rHbF production [21]. The recombinant Hbs were > 98% pure as determined by densitometry of the SDS-PAGE gel, after two steps of liquid chromatography.

**Fig. 2:**
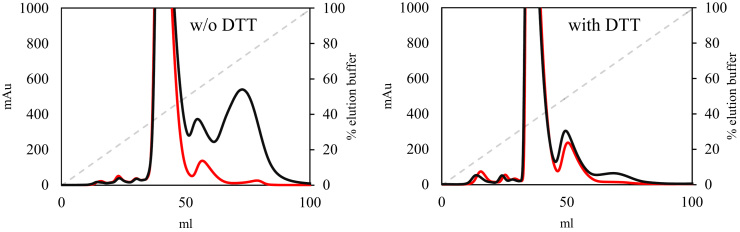
Chromatograms recorded at 280 nm, showing the gradient elution of rHbF (red) and αA19C (black) from a Hitrap Q HP column. The left chromatogram shows the elution without treatment with DTT, while the right chromatogram shows the elution after addition of DTT.

It was observed that αA19C and αA19C/γC93A, both containing additional cysteine located on the surface exposed part of the α-chain, behaved slightly differently compared to wildtype rHbF during purification. The cell lysate filtration of these two mutants was more laborious. Also, the elution profile of the second chromatography step for these mutants showed some differences. In order to verify the composition of the altered elution profile fractions, dithiothreitol (DTT) was added. When analyzed by Q HP chromatography, an elution profile similar to the one obtained with rHbF was generated (Fig. 2). The additional cysteine residues carried on the HbF mutants appear thus to be reactive. This is also supported by cysteine quantification using Ellman's reagent which showed that the γC93A mutant only generated 30% of the signal measured for rHbF, while αA19C generated twice the signal observed in rHbF. The double mutant showed a value similar to rHbF. DLS measurements were made to further investigate the size distribution of the Hb samples. rHbF showed an average size resembling previous reports of DLS measurements of Hb [28], [29], and γC93A showed similar size. The mutants containing the extra cysteine, αA19C and αA19C/γC93A, displayed larger average sizes than rHbF and γC93A, but when DTT was added the average size was decreased for these samples to values similar to rHbF and γC93A. Taken together, these results clearly demonstrate that the cysteine residues in the recombinant HbFs were reactive and the proteins show a tendency to form complexes via the cysteine residues. However, the disulfide bridges formed could easily be reduced by DTT.

Oxygen binding equilibriums were examined by generation of oxygen binding curves (OBC) in a Hemox Analyzer. All recombinant proteins successfully bound and released oxygen, and the cysteine mutations had no particular influence on the oxygen binding property of HbF (Table 1). These data are largely in agreement with a recent study comparing HbA and HbF [30].

**Table 1:** p_50_ and Hill numbers of rHbF, and the three mutants; αA19C, γC93A, and αA19C/γC93A.

Hb		p50/torr	Hill no.
rHbF	Wildtype	9.6	1.2
αA19C		9.0	1.5
γC93A		9.2	1.4
αA19C/γC93A		10	1.3

Autoxidation experiments were performed to examine the properties of the mutants compared to rHbF. The study was performed both with and without the presence of SOD and catalase. As seen in the Table 2, a slightly lower autoxidation rate was found when cysteine replaced alanine at α19 in the absence of the two enzymes. This was not observed when SOD and catalase were present, at least not in the first 24 h. On the other hand, the replacement of γCys93 to an alanine increased its autoxidation rate, in absence or presence of SOD and catalase by approximately 1.0–1.5fold, respectively.

**Table 2:** Oxidative rate data of rHbF, αA19C, γC93A, and αA19C/γC93A.

		rHbF	αA19C	γC93A	αA19C/γC93A
Autoxidation rate (h^−1^)		**0.0053** ± 0.0003	**0.0046** ± 0.0004	**0.0066** ± 0.0002	**0.0064** ± 0.0002
	+SOD and catalase	**0.0037** ± 0.0002	**0.0036** ± 0.0005	**0.0054** ± 0.0005	**0.0044** ± 0.00007
Second order rate constant: oxidation by H_2_O_2_ (µM^−1^ s^−1^)	k_γ_	**5.41·10**^**−4**^ ± 0.27**·**10^−4^	**4.99·10**^**−4**^ ± 0.19**·**10^−4^	**6.94·10**^**−4**^ ± 0.29**·**10^−4^	**6.49·10**^**−4**^ ± 0.19**·**10^−4^
	k_α_	**1.25·10**^**−4**^ ± 0.04**·**10^−4^	**1.08·10**^**−4**^ ± 0.11**·**10^−4^	**1.30·10**^**−4**^ ± 0.01**·**10^−4^	**1.33·10**^**−4**^ ± 0.08**·**10^−4^

To further study the oxidative behavior of the HbF mutants, the ferric form of the recombinant Hbs was exposed to increasing H_2_O_2_ concentrations in a stopped-flow spectrophotometer. The linear slope attained by plotting the rate constants found for rHbF and the three mutants against the H_2_O_2_ concentration gives the second order rate constant of the reaction and the results are presented in Table 2. The k_γ_ represents the oxidation of the γ-chain and the k_α_ the oxidation of the α-chain [12]. The results show that removal of the conserved cysteine at γ93 produces a higher oxidation rate of ferric Hb into ferryl form in the γ-chain, while in the α-chain no significant difference could be detected for these mutants. On the other hand, the addition of cysteine at α19 appears to indicate a slight decrease the oxidation rate in the γ-chain and α-chain in αA19C, as well as lowering the oxidation rate in the γ-chain for the double mutant αA19C/γC93A.

To test the impact of H_2_O_2_ on post-translational oxidation of oxidative hotspots in Hb, quantitative mass spectrometry was utilized to target all peptide charge states. Extracted ion chromatograms (XICs) were generated from the most abundant monoisotopic peak of each peptide isotopic profile and the resulting ratio differences were compared for oxidized and non-oxidized hotspot peptides. All amino acid oxidations identified by LC-MS/MS analysis (including Cys, Met, and Tyr residues) in this study correlated with previously published data [12], [31], [32]. With the exception of αA19C and γC93A containing peptides, the most prevalent oxidative changes were restricted to C93 containing peptides; γC93 oxidation correlated to incremental H_2_O_2_ increases in a manner consistent with previously published data [11]. Thus, the extent of tri-oxidation peroxide induced in C93 was monitored to identify how each recombinant protein responded to autooxidative/oxidative conditions. However, since γC93A and αA19C/γC93A no longer contains a cysteine at position 93, this oxidation hotspot could not be monitored (Table 3). γC93 oxidation correlates with incremental H_2_O_2_ doses. αA19C resulted in lower levels of C93 oxidation compared to rHbF; for example 10X H_2_O_2_ resulted in nearly a 2-fold lower ratio for αA19C (Table 3A). Data in Table 3B represent tri-oxidation of αC19 oxidation for the αA19C and αA19C/γC93A mutants, respectively. Substitution of cysteine into alanine at γ93 in the double mutant results in a considerable difference in αC19 oxidation – a 3.7 fold higher level than in the αA19C mutant. To further elucidate the impact of the C93A substitution on the HbF α-chain, αC104 oxidation was analyzed for rHbF, αC93A and αA19C recombinant proteins. According to the results listed in Table 3C, αC104 oxidation was low for the recombinant proteins.

**Table 3:** A) H_2_O_2_ induced tri-oxidation of Cys93 to cysteic acid in rHbF and αA19C. B) H_2_O_2_ induced tri-oxidation of αCys19 to cysteic acid in recombinant αA19C and αA19C/γC93A. C) H_2_O_2_ induced tri-oxidation of αCys104 to cysteic acid in rHbF, γC93A and αA19C.

**A**	rHbF	αA19C
Ratio H_2_O_2_/Hb	*γCys93*	*γCys93*
no H_2_O_2_	Below detection	Below detection
5X H_2_O_2_	34 ± 0.73%	23.2 ± 2.3%
10X H_2_O_2_	63.3 ± 0.13%	38.9 ± 1.2%

**B**	αA19C	αA19C/γC93A
Ratio H_2_O_2_/Hb	*αCys19*	*αCys19*
no H_2_O_2_	Below detection	Below detection
5X H_2_O_2_	5.6 ± 0.37%	23.9 ± 0.49%
10X H_2_O_2_	11.6 ± 2.3%	43.2 ± 2.5%

**C**	rHbF	αA19C	γC93A
Ratio H_2_O_2_/Hb	*αCys104*	*αCys104*	*αCys104*
5X H_2_O_2_	6.6 ± 0.11%	1.6 ± 0.31%	1.3 ± 0.15%
10X H_2_O_2_	7.0 ± 1.4%	5.5 ± 1.0%	7.3 ± 0.74%

**Fig. 3:**
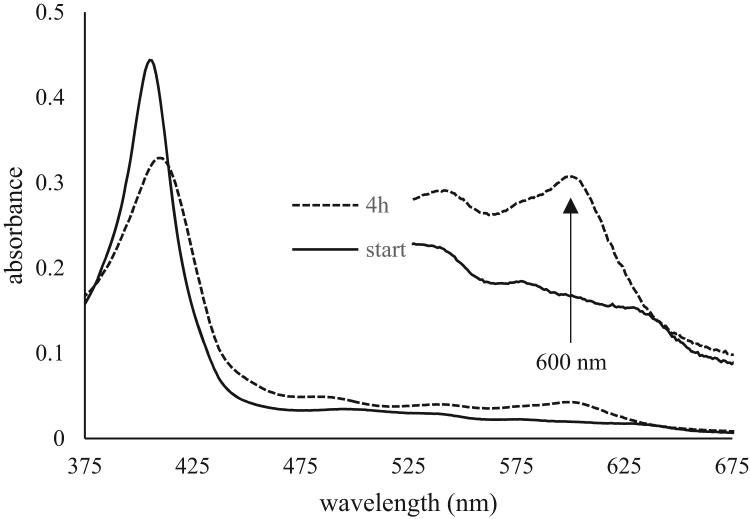
The spectra illustrates the spectral changes observed in the heme loss experiment, where the solid line represent the start spectrum and the dashed line the spectrum after 4 h. The increase at 600 nm was monitored and used to determine heme loss rates.

Heme loss in presence of excess apomyoglobin was also examined for all recombinant proteins at 1:12 Hb:apoMb ratio at 37 °C (Fig. 3 and Table 4). Heme loss from the α-chain showed no significant difference between rHbF and the three mutants. The heme loss from γ-chain on the other hand appeared to be affected by the mutations. For γC93A the rate was increased, while the αA19C exhibited a slower rate. The double mutant αA19C/γC93A showed an increased rate compared to the rHbF but not as fast as the heme loss rate for γC93A.

**Table 4:** Rate constants of heme loss from rHbF and the three mutants in presence of 1:12 excess of apoMb H64Y/V67F at 37 °C.

	**k**_**α**_**(h**^**−1**^**)**	**k**_**γ**_**(h**^**−1**^**)**
rHbF	0.397 ± 0.033	0.677 ± 0.023
αA19C	0.411 ± 0.064	0.459 ± 0.032
γC93A	0.367 ± 0.044	1.033 ± 0.043
αA19C/γC93A	0.395 ± 0.051	0.775 ± 0.014

## 4 Discussion

The conserved oxidative hotspot at position 93 in β-chain of HbA has been extensively investigated and functional roles have been assigned to βCys93, such as NO transport, ligand binding affinities, and redox reactions [11], [33], [34], [35]. Several recombinant mutants of HbA modified at βCys93 have been expressed and studied previously [33], but HbF has not received the same attention. The oxidative reactions of HbA can largely be controlled by haptoglobin, but the levels of this antioxidative protein is very low in fetal serum [36]. To clarify the role that cysteine residues play within the HbF molecule, the conserved cysteine at position 93 in the γ-chain was mutated to alanine. Storz et al. reported a decreased oxidation rate when an extra cysteine was present on the surface-exposed part of the β-chain in mouse Hb [13]. The distance between γCys93 and γ-heme iron is only 12.9 Å, well within the 19 Å distance limit that enables electron transfer on ms time scale [37], [38]. In comparison, we introduced cysteine in place of alanine at position 19 in the α-chain, at a surface exposed site far from the α-heme iron, at a distance of 22.9 Å (PDB code 4MQJ). However, electron transfer can be mediated via other residues. For instance, the more distant α19 can interact via αTyr24, located 16.5 Å from the α-heme iron and 7.9 Å from α19, as proposed previously as a route of electron transfer [22].

The rHbF mutants; αA19C, γC93A, and αA19C/γC93A were effectively expressed and purified in the same manner as the wildtype rHbF, except for addition of DTT before the Q HP column for αA19C and αA19C/γC93A. Retained structural integrity for all the mutants could therefore be assumed. The observed deviations during purification procedures for the αA19C and αA19C/γC93A mutants were hypothesized to derive from possible interactions between mutant Hb proteins or with other proteins in the *E. coli* raw extract, producing larger complexes of proteins. Addition of DTT during purification reverted abnormal chromatogram profiles back into the typical profiles normally observed in rHbF purification and the DLS measurements of the purified proteins further showed that the two mutants containing the αA19C modification exhibited larger average size before DTT was added to the protein samples. Alternative indication of successful modification in cysteine content between the wildtype rHbF and the mutants was found by quantification of cysteine residues with Ellman's reagent. Together, these findings suggest that the α19 cysteine residue is present at a surface-exposed site easily accessible for interaction with protein side chains and soluble chemicals.

The oxygen equilibrium studies proved that none of the mutations obstructed the oxygen carrying capabilities of the Hb mutants. Previously, in presence of the allosteric effector 2,3-diphosphoglycerate, a higher oxygen affinity and a lower cooperativity of the natural mutant HbF Monserrato-Sassari (γ93 Cys→Arg) have been reported [16]. The oxygen equilibrium results in this study may not support the assumption that modification of the conserved Cys93 results in a lower p_50_ and Hill number compared to rHbF, at least in the absence of 2,3-diphosphoglycerate. The natural mutant HbF Monserrato-Sassari has an arginine instead of the cysteine at position γ93. Arginine carries a large side chain, and thus may cause more structural impact than an alanine. When comparing to the HbA molecule, several β93 mutations lead to higher oxygen affinity, even when cysteine is substituted for amino acids of smaller size, and this has been attributed to destabilization of the T-state and lower binding affinity to allosteric effectors caused by weakening of a salt bridge between βAsp94 and βHis146 [33]. These amino acids are also present in HbF, γAsp94 and γHis146, but since HbF already has a lower affinity to the allosteric effector 2,3-diphosphoglycerate than HbA, partly due to γSer143 [4], the effect of substitution into an alanine at the γ93 site appears to not influence the p_50_ to the same extent as it does for β93 mutants in HbA.

The autoxidation studies showed that cysteine removal from γ93 resulted in higher autoxidation rate for both mutants harboring this mutation. This could be explained by the conserved cysteine residue acting as a superoxide scavenger and forming a thiyl radical upon HbFe^2+^-O_2_ autoxidizing to ferric HbFe^3+^ in HbA [35]. This superoxide scavenging ability consequently decreases the rate of autoxidation. Subsequently as data in Table 2 shows that a 30–50% reduction in the rates of autoxidation was seen as a result of adding SOD (superoxide ion scavenger)/catalase (peroxide scavenger) [39]. The replacement of the cysteine to alanine in this study showed that the protective mechanism was partly lost and autoxidation rates were increased. Thus, the γ93 may have the same function in HbF in terms of superoxide scavenging as β93 in HbA. Interestingly, the αA19C mutant exhibited a lower autoxidation rate compared to rHbF, but only when no SOD and catalase were present. According to Storz *et al*., autoxidation rate of mouse Hb when exposed to H_2_O_2_ was decreased when the Hb carried two cysteines in the β-chain, compared to the variant with just a single cysteine. Rerouting of oxidation patterns within a subunit may thus provide protection against an oxidizing environment. The results from the autoxidation in this study provide supplementary evidence of reorganization of electron pathways through addition of cysteine residues, not just within a single subunit, but between different subunits. This electron transfer reaction may prove particularly beneficial in an oxidizing environment.

Oxidation of the ferric form to ferryl form by H_2_O_2_ in the stopped-flow experiments showed that the γ-chain was compromised by the γC93A mutation, resulting in a higher oxidation rate in the γ-chain for those two mutants. On the other hand, the αA19C mutant again produced results indicating that oxidation rate is slightly lower, both in the γ-chain and the α-chain, compared to rHbF. Furthermore, in the double mutant αA19C/γC93A, a lower oxidation rate in the γ-chain was found. These findings additionally indicate that the cysteine on the surface of the α-chain protein provides protection from oxidative reactions in the γ-chain. Analysis of the oxidation states of different cysteine residues after exposure to oxidizing conditions with H_2_O_2_ using quantitative mass spectrometry, confirmed electron transfer from the heme vicinity of the γ-chain to the surface exposed residue α19 of the α-chain. The data obtained in this study suggest that wildtype rHbF is less oxidatively stable and resistant to autoxidation than the αA19C mutant. In previous work, it was shown that conserved Cys93 is the prominent endpoint for free-radical induced Hb oxidation, while oxidation at other residues (in β or α) is usually negligible [31], [32]. Remarkably, substitution of γCys93 to γAla93 in the double mutant results in a substantial difference in αCys19 oxidation; the 3.7-fold higher level suggests that αCys19 is potentially acting as a compensatory hotspot (assuming minimal structural perturbations) or an alternative free radical endpoint since Cys93 is no longer present. This potential for αCys19 to be a compensatory hotspot is further substantiated by the fact that γCys93 oxidation in the αA19C mutant is lower (relative to wildtype rHbF). One cysteine residue is present in the α-chain at position α104 in the wildtype rHbF molecule. Generally oxidation at this cysteine residue is negligible. Investigation whether the γC93A substitution results in a global impact on alpha subunit oxidation was performed, but as observed, αCys104 oxidation in Table 3C was low for the mutant proteins. This suggests that amino acid position α19 (and therefore αA19C) could be important as an alternative free radical endpoint in the absence of γCys93.

Heme loss studies were conducted to elucidate if the mutations produced any changes regarding possible release of heme. As free heme has been shown to be a damage-associated pattern molecule [40], the rate of heme loss from Hb is of concern regarding toxicity of Hb. The heme scavenger apoMb readily binds the dissociated heme and the rate of heme loss from Hb can be determined by monitoring spectral change in the form of a sharp peak appearing at 600 nm [26]. As heme loss is a result of excessive oxidation of Hb, the differences in oxidation rates seen in this study were anticipated to lead to heme release at different rates for rHbF and the three mutants. The results from this study showed that no difference in heme loss could be detected for α-chain for any of the four recombinant Hbs, but the heme loss from γ-chain is significantly different among all proteins. The rates obtained follow the same pattern discerned by oxidation experiments, where γC93A mutation produce a faster rate compared to rHbF, while αA19C cause a slower heme loss rate. The double mutant αA19C/γC93A exhibits a faster heme loss than rHbF, but slower than the γC93A mutant. This indicates that the oxidation rates found for the mutants directly correlates to the subsequent heme loss. The alternative hotspot that the αA19C mutation creates appears to relocate destabilizing oxidative reactions and provide some protection that also will slow down heme loss and thereby also the collapse of the protein structure.

The findings from this study can also be of interest to a more applied Hb research in the field of oxygen therapeutics. Cell-free Hb is used as the principal component but the oxidative reactions of the protein present problems that must be resolved. In this study, the focus has been on protein engineering of HbF as a substitute for HbA due to its reported potential advantages as a starting material for oxygen therapeutic development [12], [21], [22]. However, even though αA19C appears to be a promising mutation in terms of providing oxidative stability of HbF, there could also be other possible positions where cysteine residues can be introduced in the protein. This may be a way to gain further knowledge about rerouting electron transfer and the impact on oxidative reactivity of Hb, as well as other proteins.

## 5 Conclusions

As previously confirmed by studies of native HbA and recombinant mutants of βCys93, this conserved residue plays a central role in the redox reactions of Hb. From the findings presented here, there are indications that γCys93 has a role as a possible superoxide scavenger during spontaneous autoxidation. In this study, it was also established that addition of a cysteine residue in the α-chain at position α19 can influence electron transfer within the HbF molecule. This was confirmed by analyzing oxidative reactions such as rates of autoxidation and H_2_O_2_ oxidation of ferric Hb in a stopped-flow setup, as well as analyzing quantitative mass spectrometry data after H_2_O_2_ exposure. It was especially evident that the cysteine at α19 in the double mutant αA19C/γC93A showed significantly increased oxidation, acting as a compensatory hotspot when cysteine in the γ-chain was removed. αA19C mutation also contributed to a slower heme loss in the γ-chain.

The overall findings of this study are that the mutations of HbF studied produced functional proteins that exhibited normal Hb functions. The addition of cysteine at α19 produces a readily accessible site on the protein surface that is able to form di-sulfide bonds. The removal of cysteine from the γ-chain at position 93 in HbF resulted in a more oxidatively unstable protein, while addition of cysteine on the surface of the α-chain appears to provide protection from adverse oxidative reactions resulting from exposure to H_2_O_2_. Amino acid substitutions through protein engineering of the Hb molecule result in properties that may prove beneficial for an oxygen therapeutic starting material. However, the lessons learned from previous attempts need to be considered and the understanding of the multifaceted biochemistry of the Hb molecule should be extended in the strive to achieve a viable protein for oxygen delivery in clinical settings.
